# Mitochondria-associated ER membranes (MAMs) and lysosomal storage diseases

**DOI:** 10.1038/s41419-017-0025-4

**Published:** 2018-02-28

**Authors:** Ida Annunziata, Renata Sano, Alessandra d’Azzo

**Affiliations:** 1 Department of Genetics, St. Jude Children’s Research Hospital, Memphis, TN 38105 USA; 2 Division of Oncology and Center for Childhood Cancer Research, The Children’s Hospital of Philadelphia, Philadelphia, PA 19104 USA

## Abstract

Lysosomal storage diseases (LSDs) comprise a large group of disorders of catabolism, mostly due to deficiency of a single glycan-cleaving hydrolase. The consequent endo-lysosomal accumulation of undigested or partially digested substrates in cells of virtually all organs, including the nervous system, is diagnostic of these diseases and underlies pathogenesis. A subgroup of LSDs, the glycosphingolipidoses, are caused by deficiency of glycosidases that process/degrade sphingolipids and glycosphingolipids (GSLs). GSLs are among the lipid constituents of mammalian membranes, where they orderly distribute and, together with a plethora of membrane proteins, contribute to the formation of discrete membrane microdomains or lipid rafts. The composition of intracellular membranes enclosing organelles reflects that at the plasma membrane (PM). Organelles have the tendencies to tether to one another and to the PM at specific membrane contact sites that, owing to their lipid and protein content, resemble PM lipid rafts. The focus of this review is on the MAMs, mitochondria associated ER membranes, sites of juxtaposition between ER and mitochondria that function as biological hubs for the exchange of molecules and ions, and control the functional status of the reciprocal organelles. We will focus on the lipid components of the MAMs, and highlight how failure to digest or process the sialylated GSL, GM1 ganglioside, in lysosomes alters the lipid conformation and functional properties of the MAMs and leads to neuronal cell death and neurodegeneration.

## Facts

- MAMs are discrete tethering sites between the ER and mitochondria membranes with a defined composition of lipids and proteins that resembles the PM lipid rafts.
- MAMs serve as functional hubs for many physiological processes that are influenced by the specific distribution and local concentration of the lipid components (i.e. cholesterol and gangliosides)
- Lipid contents limit the recruitment and clustering of specific proteins at the MAMs, and influence their biochemical properties and functions.
- Impaired lysosomal turnover and degradation of GM1 in the LSD GM1-gangliosidosis alter the molecular composition of the MAMs and Ca2+ signaling, resulting in ER-mitochondria-mediated neuronal apoptosis.

## Open questions

- Are there other genetic defects of lysosomal catabolism that influence the function and structural characteristics of the MAMs?
- Does deregulation of signaling pathways at the MAMs, caused by their altered lipid composition, underlie aspects of pathophysiology of lipid storage diseases, especially with regard to neurodegeneration?
- Are there differences in the lipid and protein makeup of the MAMs depending on the cell types or physiological state of the cells?
- How can we monitor and assess lipid redistribution to the MAMs?

## Lysosomes and lysosomal storage diseases

The lysosome and the ubiquitin-proteasome systems converge the bulk of cellular catabolism in virtually all mammalian cells^1–3^. The dynamic interplay between these catabolic machineries counterbalances the metabolic needs of cells and ensures the homeostatic maintenance of cell physiology. Lysosomes are delegated to the compartmentalized processing/degradation/recycling of long-lived macromolecules that reach the organelles through the biosynthetic, endocytic, phagocytic or autophagic routes^3^. In addition, lysosomes have recently emerged as central platforms for specialized cellular functions, such as nutrient sensing, Ca^2+^ signaling, PM repair, cholesterol trafficking, viral/bacterial infections and cell death^2–7^. The constituents of these organelles include soluble and membrane-bound hydrolytic enzymes, lysosomal integral membrane proteins, ion channels and transporters, whose expression is regulated in turn by transcriptional and post-translational modifications that adapt their activities and functions to the cell types and the metabolic status of the cells^3, 8^. Activation of the lysosomal system may occur under both physiologic and pathologic stimuli; the result is multiform, and displays the involvement of lysosomes in such fundamental processes as metabolic regulation, nutrition, differentiation and cell defense^3,8–10^. A failure of the lysosomal system to perform its functions results in severe clinical conditions known as LSDs^9,11–13^.

The majority of LSDs are caused by deficient or defective activity of glycosidases. The natural substrates of these enzymes comprise a wide array of glycoconjugates, many of which are still unidentified, that are themselves differentially expressed and distributed in different cell types. Genetic mutations that impair their hydrolytic capacity provoke a cascade of pathologic events that are mostly triggered by the relentless accumulation of partially unprocessed/degraded substrates in these organelles, leading to the characteristic appearance of swollen lysosomes, the hallmark of these diseases. The heterogeneous composition of lysosomes and their ubiquitous distribution account for the systemic clinical manifestations of LSDs^14^.

With a combined incidence of 1:5000 live births, this group of over 70, individually rare, monogenic disorders affect multiple organs, including the nervous system^2,11^. While the genetic bases of the LSDs are relatively easy to define, the age of onset and complexity of the symptoms may depend on the type of LSD, the amount of residual enzymatic activity, the biochemical properties of the storage material, and the cell types where the storage mostly occurs^12–14^. Clinical signs may include coarse face, myoclonus, macular cherry red spot, hepatosplenomegaly, cardiac and kidney involvement and dysostosis multiplex. In addition, more than 70% of LSDs display an array of neurological abnormalities, associated with severe developmental delay, and psychiatric problems. The complexity of the phenotypes has rendered the understanding of the pathogenesis particularly challenging but also created a means for dissecting the biology of the lysosomal system and more broadly for deciphering the molecular effectors downstream of a single enzyme deficiency and accumulated metabolites.

A subgroup of LSDs linked to defective catabolism of lipids comprises the glycosphingolipidoses, NPC, and Wolman disease^15–18^. Sphingolipids and their glycosylated and sialylated derivatives, the GSLs and gangliosides, are ubiquitous and abundant components of cellular membranes in mammalian cells. Their amphipathic structure includes a basic hydrophobic membrane moiety, ceramide, composed of sphingosine and fatty acid, and a hydrophilic oligosaccharide chain that may contain one or more sialic acid residues^19,20^. They are synthesized in the ER, Golgi and trans Golgi network by the sequential addition of carbohydrate residues to their backbone ceramide; they are then transferred via vesicular and non-vesicular mechanisms to the outer leaflet of the PM^21^. Degradation of these compounds occurs along the endocytic route by the stepwise removal of individual sugars by lysosomal exoglycosidases or membrane sialidases working in concert with activator proteins^22^.

## Membrane microdomains: MAMs and their lipid raft-like characteristics

Mammalian membranes represent highly dynamic structures that maintain cell integrity and govern a myriad of cellular processes indispensable for the proper physiology of cells and tissues. PM and intracellular membranes are organized in discrete subdomains/microdomains, sharing features of lipid rafts, that are enriched in GSLs, cholesterol and ceramide. These microdomains are also known as DRMs or GEMs^23–25^. The type of lipids in these membrane microdomains limits the recruitment and clustering of specific protein components, creating functional cellular hubs for efficient (lipid) shuttling, trafficking of ions and signaling molecules^26^.

**Fig. 1:**
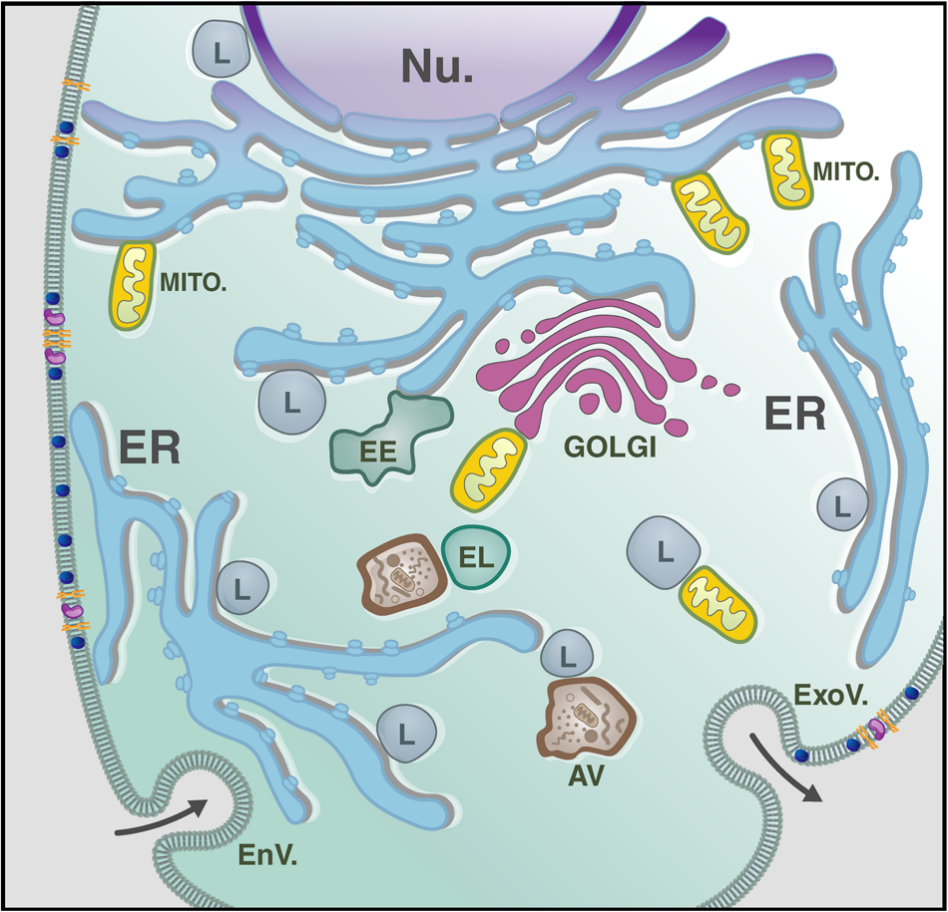
Membrane contact sites in Eukaryotes Schematic representation of an eukaryotic cell and its interorganellar membrane contact sites. The vast network of the ER participates in multiple membrane contact sites with the membranes of mitochondria (MITO.), PM, early endosome (EE), lysosome (L) and Golgi. Additionally, lysosomes can tether with mitochondrial and nuclear (Nu.) membranes. *EL* endolysosomes; *AV* autophagyc vacuoles; *EnV* endocytic vesicles; *ExoV* exocytic vesicles

**Fig. 2:**
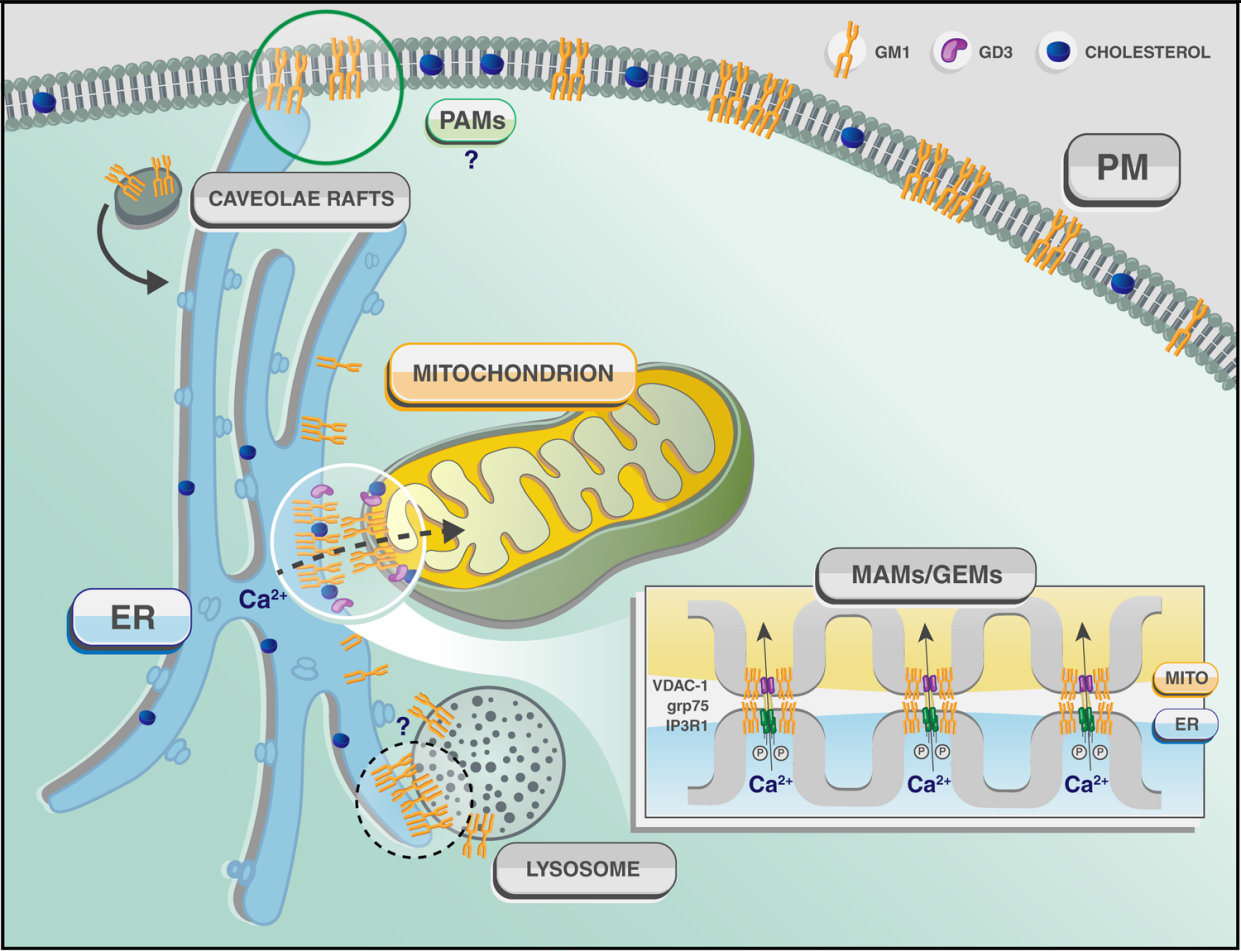
Schematic rendering of the contact sites between ER and mitochondria (MAMs), ER and PM (PAMs) and potentially ER and lysosomes (?) that explain the redistribution and buildup of GM1 in the ER membranes and the consequent activation of the apoptotic process leading to neuronal cell death in *β-gal*^*−/−*^ mice

It is now well established that all intracellular organelles are in a state of constant communication achieved at MCS^27–31^. These membrane structures create a transitory but efficient way of transferring or exchanging lipids and other macromolecules between organellar membranes. MCS are established by the tethering of membrane protein complexes and lipids, which maintain the two organelles in close proximity without undergoing fusion. Considering the widespread ER network within cells, the membranes of the ER cisternae can engage in multiple contact sites with other organellar membranes, including those of the mitochondria, the lysosomes, the Golgi apparatus, the endosomes and the PM (Fig. 1)^29,30,32,33^. These membrane microdomains are not randomly assembled but are specified by the characteristics and arrangement of the protein and lipid components they embed at either tethering membranes. This configuration allows for the compartmentalization of specific functions/activities in a controlled milieu, and explains why MCS between intracellular organelles have raft-like characteristics (Fig. 2).

So far, the best studied of these contact sites are the one formed between the membranes of the ER and the mitochondria^33–48^. These membrane microdomains, known as mitochondria associated ER-membranes or MAMs, are now established as reversible tethers that co-regulate and influence a variety of cellular processes, i.e., synthesis/transport of lipids and lipid intermediates, Ca^2+^ dynamics/signaling, autophagy, mitochondrial shape and size, apoptosis and energy metabolism^35,46–54^. These functions of the MAMs are tightly interconnected, given that changes in the topology and concentration of their constituents can dramatically alter their homeostatic control of specific ER and mitochondrial functions with pathological effects that can lead to cell demise.

To date, there is still little understanding of the way the turnover of membrane lipids, particularly GSLs and cholesterol, by lysosomal hydrolases can modulate the local concentration of these constituents at the MAMs and, in turn, their function. However, the prediction is that defective lysosomal catabolism of lipids, causing the buildup of these compounds at the PM and in lysosomes, may provoke their redistribution to the limiting membranes of other organelles and eventually alter the lipid composites of MCS. In this review, we will give a specific example of how impaired degradation of the sialylated GSL, GM1, due to deficiency of the lysosomal enzyme β-Gal (Table 1) in the neurodegenerative LSD GM1-gangliosidosis, provokes the redistribution of this ganglioside to the ER membranes, which, in turn, changes its local concentration within MAMs and alters MAM’s functions (Fig. 2).

**Table 1:** List of proteins cited in this review, with their localization, function, and main interactors

Protein	Localization	Function	Known interactors	References
β-galactosidase	Lysosome	Catalyzes the hydrolysis of a terminal β-linked galactose residue from ganglioside substrates and other glycoconjugates	Protective protein cathepsi A (PPCA), neuraminidase 1 (NEU1)	^126, 127^
Cathepsin D	Lysosome	Aspartic-type endopeptidase activity		^80^
Palmitoyl-protein thioesterase 1, PPT1	Lysosome	Removes thioester-linked fatty acyl groups such as palmitate from cysteine residues	CLN5	^80^
Tripeptidyl peptidase 1, TPP1	Lysososome	Cleaves N-terminal tripeptides from substrates, and has weaker endopeptidase activity	CLN5	^80^
PSS1	ER face of the MAMs	Phosphatidylserine synthase 1		^128^
PSS2	ER face of the MAMs	Phosphatidylserine synthase 2		^128^
PS- decarboxylase	Mitochondrial side of the MAMs	Phosphatidylserine- decarboxylase		^59^
PE-methyltransferase, PEMT	MAMs	Phosphatidylethanolamine-methyltransferase		^62^
Mdm10	Mitochondria/MAMs	ERMES complex	Mdm34, Mmm1, Mdm12, Gem1	^45, 71^
Mdm34	Mitochondria/MAMs	ERMES complex	Mdm10, Mmm1, Mdm12, Gem1	^45, 73^
Mmm1	ER/MAMs	ERMES complex	Mdm34, Mmm10, Mdm12, Gem1	^45, 72^
Mdm12	Cytosol/MAMs	ERMES complex	Mdm34, Mmm10, Mdm1, Gem1	^45, 73^
Lam6	MAMs, vacuole-mitochondria patches, nuclear-vacuole contact sites	ERMES complex, vCLAMP (vacuole and mitochondria patch), and NVJ (nuclear vacuolar junction)	Mdm10, Mmm1, Mdm12, Mdm34, Vps39, Nvj1, Vac8	^74, 75^
Gem1	MAMs	ERMES complex	Mdm10, Mmm1, Mdm12, Mdm34	^70, 72^
EMC1	MAMs	EMC, PS transfer, MAMs architecture	TOM5	^78^
EMC2	MAMs	EMC, PS transfer, MAMs architecture	TOM5	^78^
EMC3	MAMs	EMC, PS transfer, MAMs architecture	TOM5	^78^
EMC4	MAMs	EMC, PS transfer, MAMs architecture	TOM5	^78^
EMC5	MAMs	EMC, PS transfer, MAMs architecture	TOM5	^78^
EMC6	MAMs	EMC, PS transfer, MAMs architecture	TOM5	^78^
CLN8	ER integral membrane protein	Transmembrane protein belonging to a family of proteins containing TLC domains, which are postulated to function in lipid synthesis, transport, or sensing.	CLN5	^80^
Caspase 12	ER	Member of the cysteine-aspartic acid protease family responsible for ER-stress-induced apoptosis		^102, 129^
PC- cytidyltransferase, CTP	Cytosol, MAMs	Involved in the regulation of phosphatidylcholine biosynthesis		^86^
Acetyl-CoA:cholesterol acyltransferase, ACAT	MAMs	Catalyzes the reversible formation of acetoacetyl-CoA from two molecules of acetyl-CoA		^35, 90^
Steroidogenic acute regulatory protein, StaR	MAMs, mitochondria	Plays a key role in the acute regulation of steroid hormone synthesis by enhancing the conversion of cholesterol into pregnenolone	TOMM22, VDAC2	^97^
Sigma-1 receptor	ER face of the MAMs	Interacts with a variety of psychotomimetic drugs, including cocaine and amphetamines. The receptor is believed to play an important role in the cellular functions of various tissues associated with the endocrine, immune, and nervous systems.	BiP, IP3R3	^100^
VDAC-1	Mitochondrial side of the MAMs	Facilitates the exchange of metabolites and ions across the outer mitochondrial membrane	Grp75	^130^
IP3R-1	ER, MAMs	Mediates calcium release from the endoplasmic reticulum following stimulation by inositol 1,4,5-trisphosphate	GRP75	^52, 130, 131^
GRP75	ER, mitochondria, MAMs	Member of the heat shock protein 70 gene family, functions as scaffold between IP3R-1 and VDAC1	IP3R-1 and VDAC1	^52, 130^
BiP	ER, MAMs	Involved in the folding and assembly of proteins in the ER	Interacts with many ER proteins	^108, 132^
Calnexin	ER, MAMs	Ca^2+^-binding protein that interacts transiently with newly synthesized N-linked glycoproteins, facilitating protein folding and assembly	PACS2, AMBRA1, WIPI1	^116, 133^
hFis1	Mitochondria	Promotes mitochondrial fission	t-Bid, Bax,, VDAC1	^134, 135^
t-Bid	Mitochondria	Member of the BCL-2 family of cell death regulators	Eterodimerizes with either agonist BAX or antagonist BCL2, VDAC1	^134, 136^
Bax	Mitochondria	Member of the BCL-2 family of cell death regulators	BCL2 family members form hetero- or homodimers and act as anti- or pro-apoptotic regulators	^134, 137^
LC3	Autophagic vacuoles	LC3 (encoded by MAPLC3A and MAPLC3B) is the homolog of the yeast ATG8, an important marker and effector of autophagy	Microtubules, FYCO1, TP53INP1 and TP53INP2, TBC1D25, SQSTM1, ATG4B, MAPK15 and BNIP3, MAPB1, KEAP1, PCM1, OFD1, CEP131, TECPR2,TBC1D5, UBQLN1, UBQLN2, UBQLN4, UBQLN1, ATG13, FAM134A, FAM134B, FAM134C	^140–152^
AMBRA1	Autophagic vacuoles	Regulates autophagy and development of the nervous system	BECN1, BECN2, BCL2, dynein light chains 1 and 2, WIPI1, calnexin	^116, 151, 152^
WIPI1	Autophagic vacuoles	Plays an important role in autophagy and in particular starvation- and calcium-mediated autophagy	Interacts with androgen receptor (AR) and the estrogen receptors ESR1, and ESR2, calnexin	^116, 153^
CHOP	Nucleus	CCAAT-enhancer-binding protein homologous protein		^102^
JNK2	Cytosol	JNKs (c-Jun N-terminal kinases) are a group of mitogen-activated protein kinases activated by various environmental stresses		^102, 154^

## Lipid synthesis at the MAMs

### Phospholipids

Since the discovery of the MAMs and the identification of their role in maintaining intracellular Ca^2+^ homeostasis^35,36,47,49,52,55^, a large body of experimental work has implicated these specific microdomains in both physiological and pathological processes. One of the fundamental functions of the MAMs is the coordinated biosynthesis of phospholipids and their transport^35^. This is because, beside the ER, other intracellular organelles, including the mitochondria, lack the ability to synthesize de novo their phospholipids and rely on the ER as their only source^56^. On these premises, the close proximity of the ER and the mitochondria at the MAMs (~10–30 nm) provides an insulated environment against the hydrophilicity of the cytosol, and favors the bi-directional, non-vesicular lipid transfer at these microdomains^57^. This exchange in trans is evidenced in the metabolism of PS and PC, that has been shown to occur in part at the MAMs^35,58^. In mammalian cells two PS synthases, PSS1 and PSS2 (Table 1), are present at high concentrations in the MAMs, where they produce PS by base-exchange of the head groups of PC or PE with serine in a Ca^2+^ dependent manner^58^. These reactions promote the release of Ca^2+^ into the ER, an ATP dependent process^35,58^. PSS1 and PSS2 localize at the ER face of the MAMs, opposite to the PS-decarboxylase (Table 1), which is located at the mitochondrial side^59^. Their strategic position creates a gradient of PS concentration that facilitates the transfer of PS from the MAMs to the mitochondrial inner membrane. The latter is a rate-limiting step for PS conversion into PE by the decarboxylase. Alternatively, PE is generated by acylation of lyso-PE, a reaction that also occurs in the MAMs^60–62^. Within these contact sites PE can then undergo conversion into PC by three consecutive methylation reactions catalyzed by the methyltransferase PEMT (Table 1), also localized at the MAMs^62,63^. This set of enzymatic reactions ensures the controlled supply of phospholipids to the mitochondria, which is necessary for maintaining mitochondrial membrane integrity, especially following fusion and fission of the organelles^64–66^. Notably, the ablation of either PSS1 or PSS2, but not both enzymes, in mice is compatible with life, despite the reduction of serine exchange activity and overall tissue reduction of PS^67^; instead, silencing of PS-decarboxylase reduces mitochondrial PE content, and results in mitochondrial dysfunction and lethality in embryos^68^.

The active transfer of phospholipids by multiprotein complexes has also been shown to occur at the MAMs, although so far only in the budding yeast and not in mammalian cells. In yeast, genetic screenings have identified macromolecular protein complexes that tether the ER and mitochondrial membranes at contact sites, and possibly coordinate the trafficking of phospholipids and phospholipid intermediates between the two organelles. One of these complexes, named ERMES^45,65,69^, (Table 1) was found by screening for yeast mutants that failed to grow unless they expressed a synthetic fusion protein that artificially created contact sites between the two organellar membranes. ERMES comprises the two mitochondrial outer membrane proteins Mdm10 and Mdm34, the integral ER membrane protein Mmm1 and the cytosolic protein Mdm12^45,70–73^. In addition, Gem1, the yeast ortholog of the mitochondrial Rho GTPases 1 and 2, Miro1 and Miro2^70^, and more recently Lam6^74,75^ (Table 1), have been shown to be part of and to regulate the ERMES complex. It is noteworthy that Lam6 contains a START domain (see below) and has the ability to bind and transfer sterol across intracellular contact sites^76^; hence, Lam6 may exert a similar task within the ERMES complex. However, while the role of this complex in tethering ER and mitochondria has been demonstrated, its function in phospholipid transport remains controversial because of apparently contradictory results reported in the literature. In one study, ERMES was shown to mediate lipid exchange between ER and mitochondria^45^, while in another study the transport of PS between these organelles was shown to be ERMES- and Gem1-independent^77^. These authors argued that the altered lipid profiles of cells lacking ERMES^45^ were caused by defects in mitochondrial morphology, rather than in phospholipid metabolism^77^. Furthermore, all components of the ERMES complex, with the exception of Gem1 and Lam6, lack obvious homologs in higher eukaryotes; for this reason it is still under debate whether an ERMES-like complex exists in mammalian cells^70^.

Recently, an array-based genetic interaction screen, aimed to discover genes required for phospholipid exchange at the MAMs^78^, revealed that yeast mutants missing multiple proteins of another complex, EMC (Table 1), had defects in PS transfer to mitochondria, reduced number of contact sites and compromised mitochondrial function. The EMC complex contains six conserved proteins, EMC1–6, and interacts with TOM5^78^. This large multiprotein complex represents another tethering structure between ER and mitochondrial membranes that transfers phospholipids independently of ERMES^78^. However, while Lam6 and the EMC proteins do have metazoan orthologs, it is still unclear whether these orthologs effectively function as regulators of ER-mitochondria contact sites in mammalian cells, and whether they facilitate lipid shuttling. In addition, we cannot exclude that physical flipping of the lipids may occur between the juxtaposed membranes if they are in sufficiently close proximity.

### Defective phospholipid synthesis in the MAMs of Cln8 deficient mice

Interestingly, already two decades ago Vance et al^79^. examined the role of the MAMs in the metabolism of phospholipids, particularly PS, using the mouse model of the motor neuron disorder, neuronal ceroid lipofuscinosis or NCL. NCLs, also referred to as CLNs^80^, are a group of at least 14 congenital neurodegenerative disorders with onset in infancy, adolescence, or young adulthood, depending on the gene involved and the severity of the clinical symptoms^81^. These diseases are characterized by progressive psychomotor deterioration, epilepsy, and blindness^81^. Their histopathology hallmark is the accumulation in neurons and other cell types of the autofluorescent lipopigment, lipofuscin^82^. NCLs are caused by mutations in different genes, encoding soluble lysosomal enzymes (i.e., cathepsin D, PPT1, TPP1) or (non-lysosomal) integral membrane proteins (Table 1)^80^. One example of the latter group is CLN8, a transmembrane protein that is localized in the ER membranes or shuttles between the ER and Golgi compartments, and is thought to take part in lipid synthesis and transport^83^. Deficiency of CLN8 is linked to one of the late infantile variants of NCLs, and is associated with increase in glycerophospholipids containing polyunsaturated fatty acyl chains, such as PS and PE, and reduced concentrations of ceramide, galactosyl- and lactosylceramide, sulfatides and fatty acyl chain-containing molecular species^84^. A homozygous mutation in the orthologous mouse gene (Cln8) was identified in a naturally occurring NCL model, the motor neuron degeneration (mnd) mouse^85^. Using MAMs isolated from the liver of these animals, Vance et al^79^. demonstrated that the levels and activity of PEMT2 and other two key enzymes for the synthesis of phospholipids (CTP and PS synthase)^86^(Table 1) were greatly reduced. Although these reduced activities did not hamper the import of phospholipids into the mitochondria nor the PS metabolism, other proteins normally present in mitochondria partially redistributed to microsomes, suggesting intrinsic defects of the MAMs in the mutant mice^79^. Nearly 10 years later, by analyzing the neurodegenerative events occurring in specific brain regions of the same mouse model at different stages of disease progression, two groups of investigators reported variable activation of effectors of ER-stress-mediated apoptosis and inflammation^87^, as well as defective mitochondria buffering capacities of neurons^88^. Together these studies support the idea that deregulated activity/function of the MAMs relative to lipid synthesis and transport could be a major contributor of neurodegeneration and disease progression in CNL8 deficiency.

### Cholesterol and ceramide

Other essential lipids at the mitochondrial membranes are cholesterol and ceramide (Fig. 2). As it is the case for the phospholipids, cholesterol and ceramide are imported into the mitochondria where they serve as structural components of the membranes or as precursors for the synthesis of steroid hormones^89^. These lipids are produced at least in part in the MAMs, and this accounts for the activities of the cholesterol synthetic enzymes and ceramide synthase being higher in the MAMs than in the ER or mitochondria, respectively^57,64^.

Basal storage of cholesterol in the form of cholesteryl esters is maintained predominantly through the activity of ACAT (Table 1)^90^. This enzyme catalyzes the esterification of insoluble, membrane-bound cholesterol into cholesteryl ester, which is subsequently incorporated into lipid droplets, thereby preventing the buildup of toxic cholesterol at the membranes. ACAT and its enzymatic activity are enriched in MAMs, and in fact, this enzyme has been used as a reliable marker of these microdomains^35^. Under stress or other pathological conditions, the flux of cholesterol may change dramatically due to either augmented hydrolysis of stored cholesteryl esters, increased uptake of plasma cholesterol, or transport of free cholesterol into the mitochondria. The ensuing accumulation of cholesterol within the MAMs is at the basis of severe disease conditions including Alzheimer’s disease, steatohepatitis, and cancer^91–94^, each of whom have been linked to altered signaling pathways occurring at the MAMs.

Similarly, changes in ceramide levels in the MAMs may occur under pathological stimuli. For instance, upon radiation both ceramide synthesis and ceramide levels increase exclusively in the MAMs, but not in the ER and the mitochondria^95^, albeit the implications of this phenomenon are unknown. We can postulate, however, that the increased pool of ceramide in the MAMs creates a reservoir of this lipid, which can be readily transferred into the mitochondria for the initiation of an apoptotic process, if the pathogenic insult persists. Together these observations suggest that enrichment of lipid synthetic enzymes at the MAMs may serve the purpose of compartmentalizing the transfer of lipid pools into the mitochondria, thereby limiting the toxic effects of lipid overload in this organelle.

Cholesterol is not only synthesized in the MAMs, but also uses these microdomains for its transport into the mitochondria^96^. This process occurs under acute stress condition or when there is the need for increased cholesterol transfer into the mitochondria, for instance during the synthesis of steroid hormones. The latter process is catalyzed by the sequential action of a set of steroidogenic enzymes, one of which, StAR (Table 1), is rate limiting for the subsequent biosynthetic reactions^96^. As mentioned earlier, StAR contains a START domain that is required for the binding to and transport of cholesterol. The mode of activation of this enzyme is unusual because the 37 kDa StAR precursor is first translocated into the MAMs, where it binds cholesterol and interacts with VDAC2, a prerequisite for its processing into a 32 kDa intermediate^97^. This form is then imported into the mitochondrial matrix as a fully active 30 kDa cholesterol-bound protein by a complex formed by TOMM22 and VDAC2 (Table 1)^97^. VDAC2 appears to be a key player in this process, because, in its absence, the amount of mitochondrial StAR is reduced and the protein cannot be efficiently loaded onto the MAMs. Consequently, a reduced amount of cholesterol is imported into the mitochondria, which eventually affects the synthesis of steroids. Also in this example, the MAMs act as functional hubs for the pathways of cholesterol transport and steroidogenesis^97^.

The actual lipid composition of the MAMs has gained special attention in recent years^52,98,99^. Several studies have revealed that specific types of lipids are embedded and concentrate in these microdomains, rendering the composition of the tethering membranes topologically different from that of the reciprocal ER and mitochondrial membranes. The gathering of lipids at the MAMs, and the modulation of their local concentration, change the structure of these contact sites and their ability to recruit specialized protein assemblies, ultimately altering the MAMs’ physiological functions and activities. For example, sigma-1 receptor, a structural component of the MAMs^100^ that participates in Ca^2+^ signaling, associates within the raft/GEM fraction of the MAMs with ceramide and cholesterol^98,99^. The high levels of cholesterol within the MAMs, which is 5–7 times higher than that in the ER, and the accumulation of ceramide at the same sites have been shown to cause partitioning of sigma-1 receptor to the raft/GEM subdomain of the MAMs^98^. Instead, depletion of either lipid from the MAMs relocates the sigma-1 receptor from the MAMs to the ER membrane, likely altering Ca^2+^ signaling^99^. It was also demonstrated that reducing cholesterol concentration within the MAMs with MBCD, a circular oligosaccharide used to extract cholesterol from membranes^98^, significantly increases MAMs copurification with mitochondria, suggesting that cholesterol may also act as a negative regulator of MAMs formation.

## Sialylated glysoshingolipids: gangliosides

**Fig. 3:**
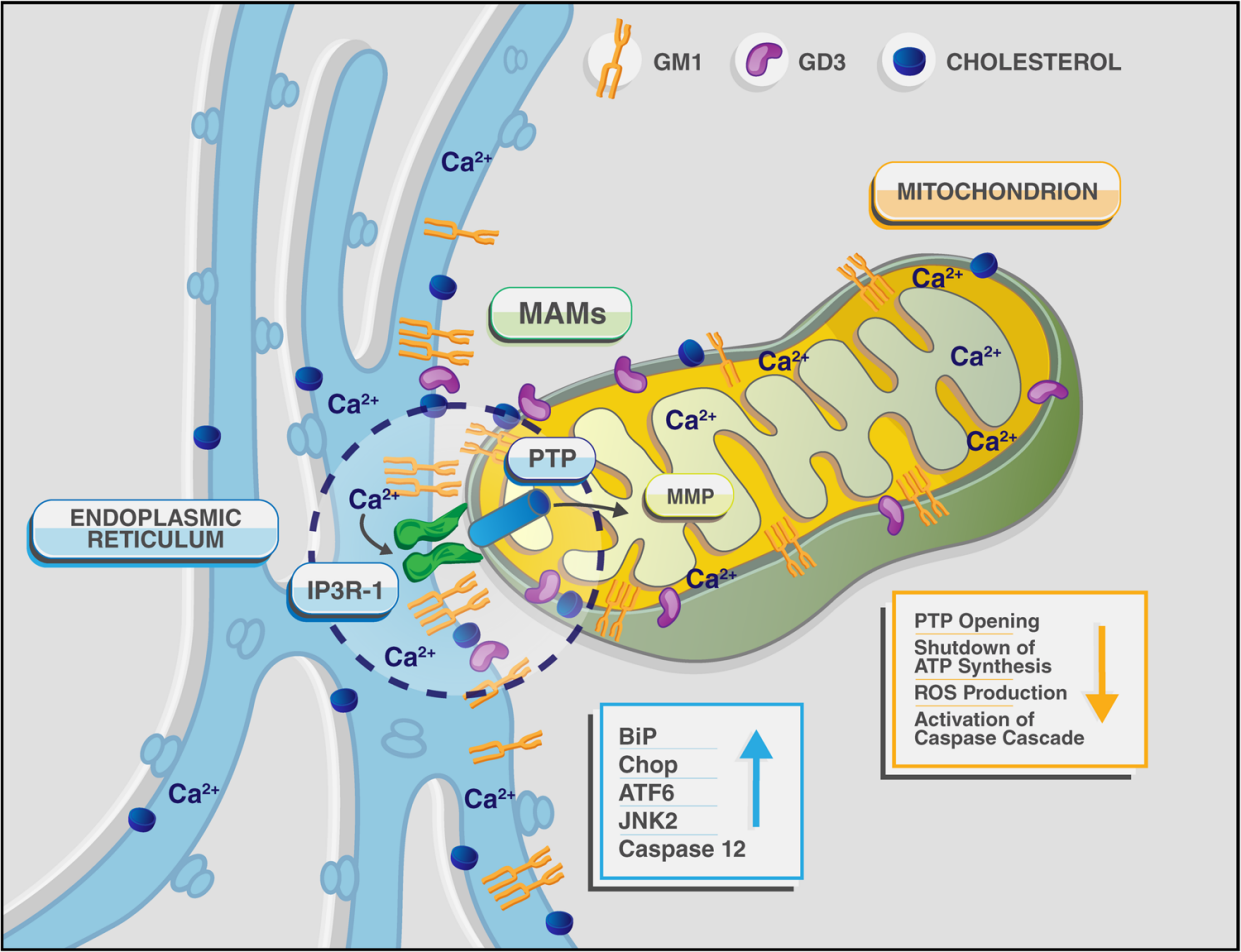
Schematic representation of a single MAM, depicted as a functional hub for the aberrant transfer of Ca^2+^ between ER and mitochondria, leading to ER- and mitochondria- mediated neuronal cell death in GM1-gangliosidosis The figure also lists the principal effectors of the apoptotic process described in Tessitore et al., 2004^108^ and Sano et al., 2009^52^

Other quantitatively minor, but important lipids for the function of the MAMs, are the gangliosides, the major class of acidic GSLs, containing one or more sialic acid residues. Gangliosides are structural components of the outer leaflet of PM and the nuclear envelop, where they segregate, together with cholesterol and other sphingolipids, into raft microdomains^102–104^. They are particularly abundant in the nervous system, where they account for approximately 10–12% of the total lipid contents of neuronal membranes^102–104^, whereas they occur at relatively low levels in other tissues. Their diversity and structural complexity suggest that gangliosides are not biologically redundant, but have unique functions. Beside exhibiting receptor or co-receptor function for cytokines, toxins, viruses and bacteria^19,105–107^, gangliosides are key signaling molecules that take part in biological processes as pivotal as cellular recognition and adhesion, receptor signal transduction, Ca^2+^ signaling, growth regulation, and differentiation^101,103,107^. Gangliosides are also important messengers of the adaptive responses to stress, including apoptosis. Under stress conditions that cause an increase of their intracellular concentration, gangliosides can tilt the homeostatic balance towards the induction of an apoptotic program (Fig. 3)^52,108^. In addition to their role at the PM, there is now experimental evidence that gangliosides can reside at the MAMs, where their effect on often opposite cell fate decisions again depends on their local concentration, structural characteristics and sugar modifications. However, it is not yet clear whether gangliosides directly perturb membrane composition and permeability, or they influence the recruitment/function of membrane proteins.

### MAM’s localized GD3 and autophagy

The disialogaglioside GD3 has been first described as a tumor associated antigen, because it was found overexpressed in multiple tumors^109,110^. Since those early studies, GD3 has been shown to induce mitochondrial apoptosis in several cell types, including human hematopoietic cells, epithelial cells and neural cells^111,112^. GD3 appears to exert this function when localized in the raft/GEM microdomains of the mitochondrial membrane (Figs. 2 and 3), where it associates with VDAC1 and the fission protein hFis1 to form a structural complex that recruits two of the Bcl-2 family of proteins, t-Bid and Bax (Table 1). Once formed, this multimolecular complex triggers mitochondria depolarization and apoptosis. In support of these findings is the observation that disruption of lipid microdomains in isolated mitochondria by MBCD rescues the mitochondria depolarization induced by GD3^113^.

Besides its role in apoptosis, recently GD3 has also been implicated in the formation of autophagosomes, because it was shown to physically interact with MAP1LC3/LC3 (Table 1)^114,115^. In a follow-up study, the same group has demonstrated that specifically in the raft/GEM fraction of the MAMs, GD3 is associated with two important effectors of autophagosome’s formation, AMBRA1 and WIPI1 (Table 1)^116^. These authors further showed that under starvation-induced autophagy the concentration of GD3 increases within the microdomains, which favors the interaction of this ganglioside with the chaperon calnexin, another resident protein of the MAMs that interacts with AMBRA1 and WIPI1. Thus, following autophagic induction, an increased amount of GD3 clusters together with AMBRA1, WIPI1 and calnexin in the lipid-enriched fraction of the MAMs; these results point to an active role for this ganglioside in the early phases of the autophagic process, specifically at these membrane microdomains. In contrast, reducing the levels of GD3 by inhibition of ganglioside synthesis hinders the initiation of autophagy^116^. Thus, as it is the case for other lipids, modulation of the gangliosides’ concentration in the MAMs greatly influences the coordinated regulation of autophagy^116^.

### GM1-gangliosidosis: GM1 at the MAMs and neurodegeneration

Another sialylated GSL identified as a structural component of the MAMs, albeit present at very low concentration under physiological conditions, is GM1. GM1 is one of the most abundant gangliosides present in the adult neuronal membranes, and is the only ganglioside shown to bind Ca^2+^ and to modulate Ca^2+^ flux across membranes (Figs. 2 and 3)^52,102,108,117,118^.

Accumulation of GM1 above its physiological threshold occurs in GM1-gangliosidosis, a generalized, neurodegenerative LSD caused by deficiency of the GM1-cleaving enzyme β–Gal^119^. This very severe condition affects primarily infants, but milder cases, with a longer survival, occur in adolescents and adults^119^. The early onset form of GM1-gangliosidosis presents with growth retardation, progressive neurologic deterioration, due to extensive brain atrophy, visceromegaly, and skeletal dysplasia. Abnormal amounts of GM1 and, to a lesser extent its asialo-derivative GA1, accumulate in the brain, and oligosaccharides derived from glycoproteins and keratan sulfate are excreted in the urine. *Beta-Gal*^*–/–*^ mice, an authentic model of GM1-gangliosidosis^120^, develop a severe, generalized nervous system disease, resulting in tremors, ataxia and abnormal gait, which culminates with rigidity and paralysis of the hind limbs^120^.

**Fig. 4:**
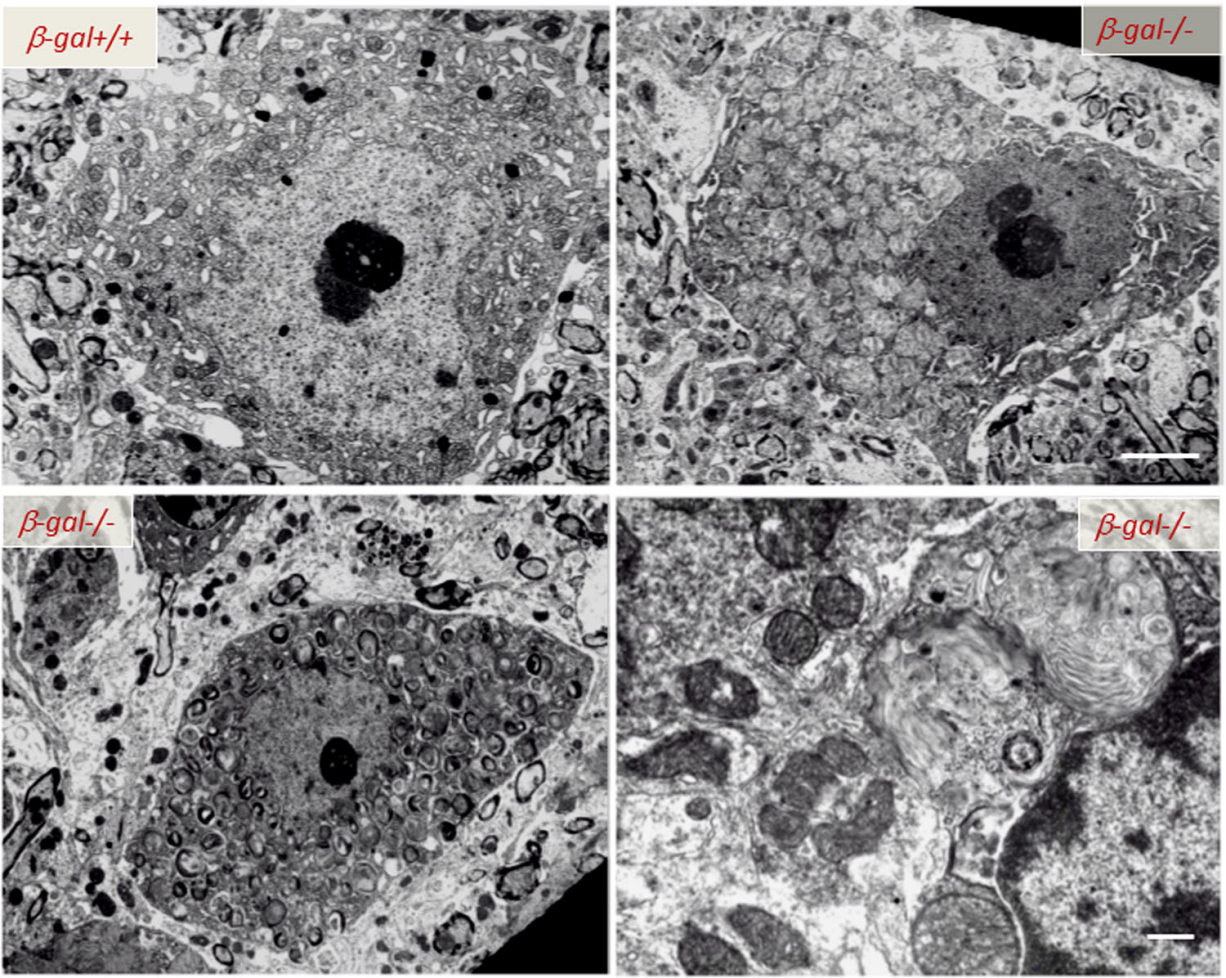
Ultrastructural abnormalities in the CNS of *β-Gal*^*−/−*^ mice Transmission electron microscopy of spinal cord neurons from 3-month-old *β-Gal*^*−/−*^ and *β-Gal*^*+/+*^ mice shows evidence in the affected mouse of an expanded lysosomal compartment with enlarged lysosomes filled with membranous material due to accumulation of GM1-ganglioside. Scale bars: 1 μm; lover right panel 0.5 μm. Adapted from the original article Tessitore et al., 2004^108^ with the permission of Elsevier

Neurons, the primary affected cells of the disease, undergo massive, age-dependent accumulation of both GM1 and GA1^120^. This phenotype is accompanied by profound histopathological changes throughout the CNS, associated with progressive expansion of the lysosomal compartment as the animals age (Fig. 4). Degenerating neurons filled with lysosomes containing membranous material and remnants of the ER and ribosomes are emblematic of the disease (Fig. 4). Neuronal cell death is followed by micro- and astrogliosis, a sign of an elicited widespread CNS inflammation^121–123^. In *β-Gal*^*–/–*^ mice impaired lysosomal degradation of GM1 in neurons is accompanied by the abnormal redistribution of this ganglioside from the PM and lysosomes to the ER membranes (Fig. 2). Studies seeking for a pathogenic mechanism that would link the neuronal cell death to the primary storage product, revealed that the abnormal GM1 levels at the ER membranes led to depletion of the ER Ca^2+^ store, followed by activation of an UPR^123^. Initially elicited as survival mechanism via upregulation of ER-resident chaperones and folding catalysts, such as BiP (Table 1), and inhibition of protein synthesis^124^, prolonged UPR activation under a persistent pathologic stimulus causes permanent damage and triggers an ER stress-mediated apoptotic program^125^. In *β-Gal*^*−/−*^ brain and spinal cord, upregulation and activation of the UPR molecular effectors responsible for cell survival (e.g., BiP) is associated with increased levels of the transcription factor ATF6, the proapoptotic mediator CHOP, the phosphorylated form of the kinase JNK2, and the cleaved, active form of caspase-12, and culminates with apoptosis (Fig. 3 and Table 1)^108^. Activation of the UPR appeared to be directly downstream of GM1 accumulation in the ER membranes, because reducing the levels of de novo synthesized GM1 reverted UPR activation^108^.

**Fig. 5:**
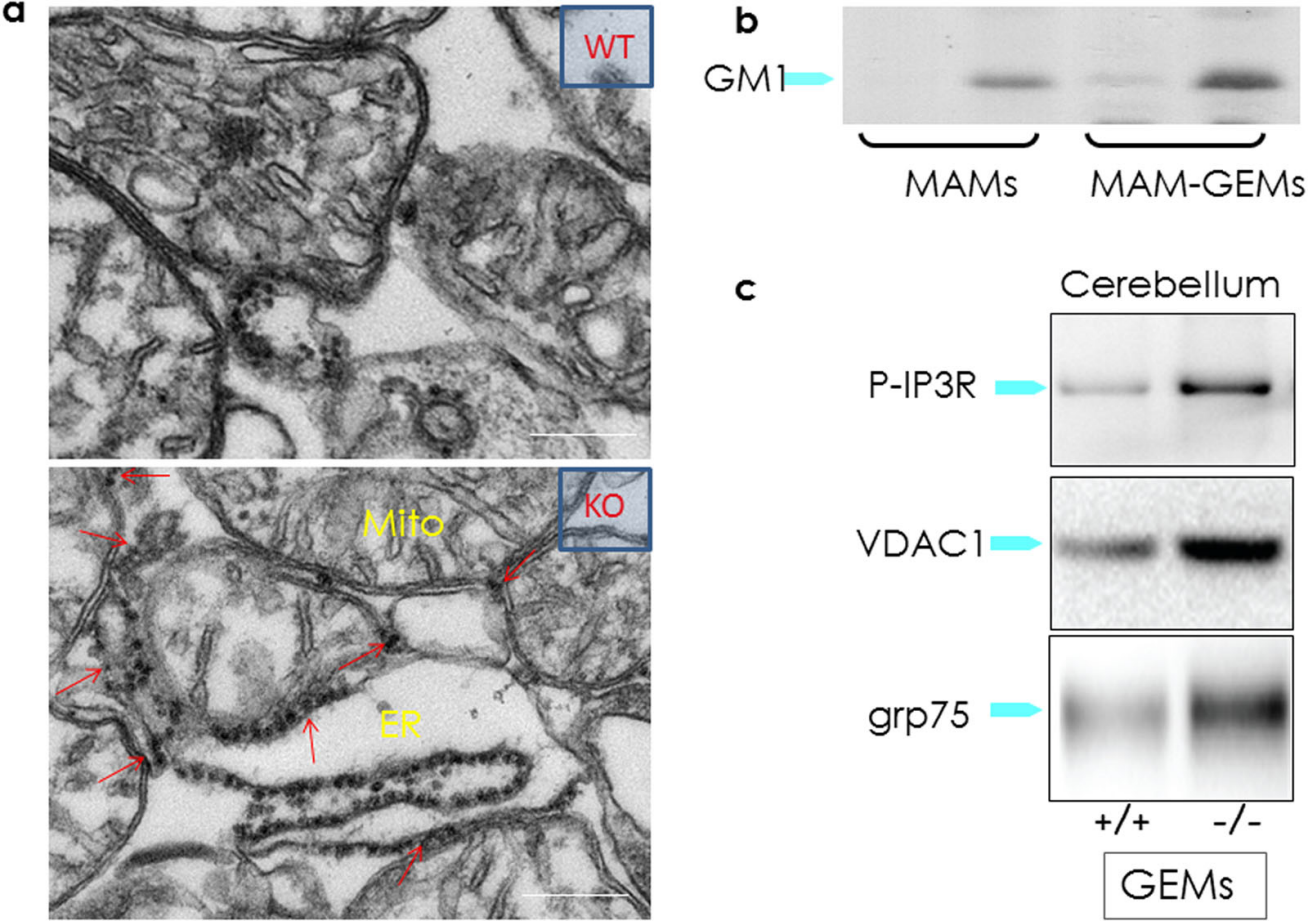
GM1 accumulation in the GEMs alters MAMs dynamics **a** Representative electron micrographs of mitochondria isolated from *β-Gal*^+/+^ and *β-Gal*^−/−^ brains showed larger areas of juxtaposition between ER and mitochondria in the *β-Gal*^−/−^ preparations compared to the WT. **b** TLC analysis of lipids from the purified MAMs, and the Triton-extracted (Triton extr. MAMs) and Triton-insoluble fractions (GEMs) of the MAMs demonstrated the buildup of GM1 in all *β-Gal*^−/−^ fractions. **c** Increased levels of phosphorylated IP3R1, VDAC1, and GRP75 were detected in the GEMs extracted from *β-Gal*^−/−^ brains compared to *β-Gal*^+/+^ brains. Adapted from the original article Sano et al., 2009^52^ with the permission of Elsevier

**Fig. 6:**
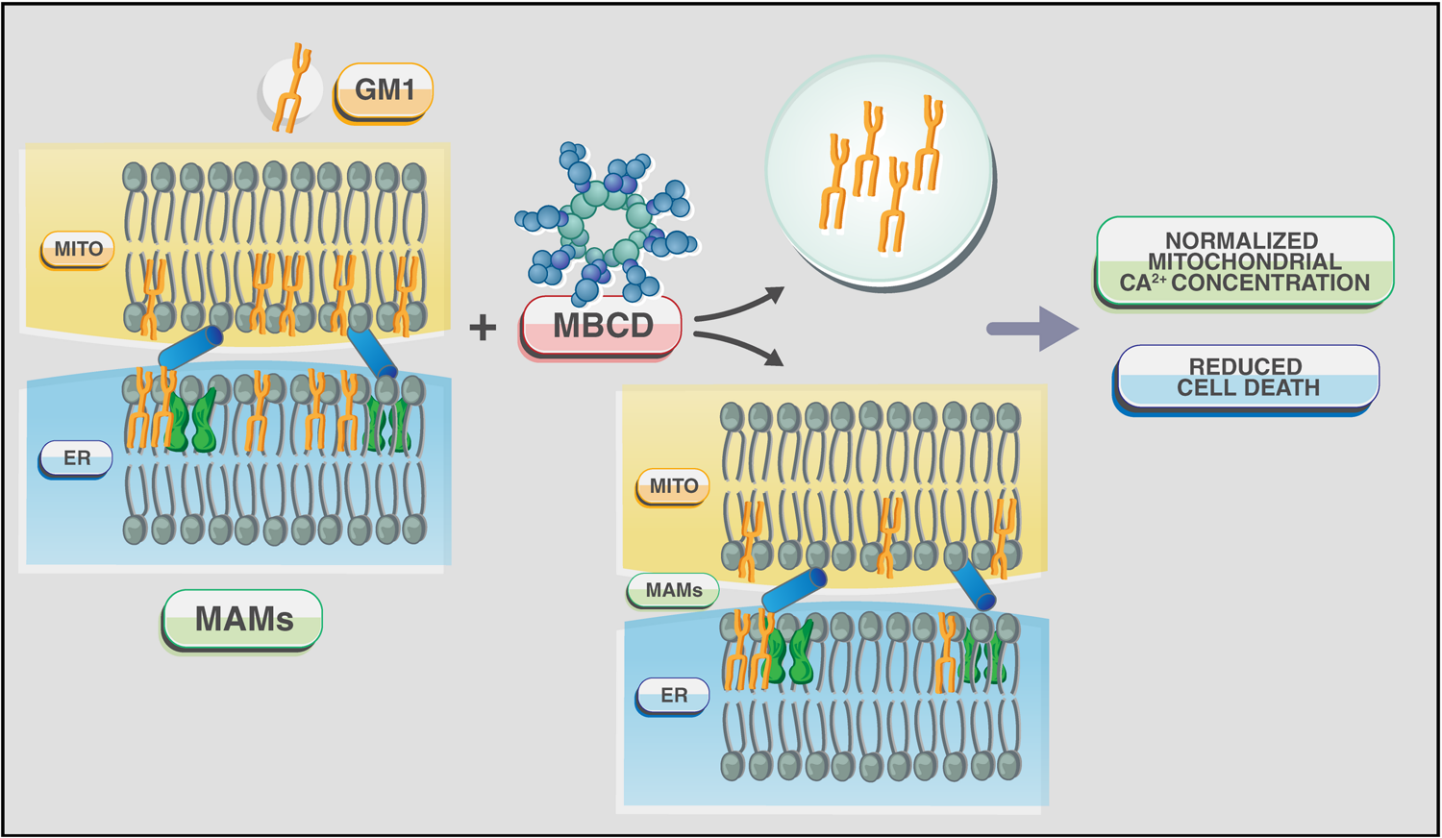
Schematic rendering of the effects of MBCD on MAMs/GEMs in GM1-accumulating cells MBCD efficiently extracts GM1 from these microdomains and, in turn, reverts mitochondrial Ca^2+^ overload and apoptosis

The intrinsic ability of GM1 to bind Ca^2+^ and its redistribution in the ER membranes suggested that this ganglioside could reside also in the MAMs. At this location, GM1 accumulation could perturb the topology of the microdomains and their Ca^2+^ buffering capacity. Transmission electron microscopy analyses of crude mitochondria isolated from *β-Gal*^*–/–*^ mouse brains indeed identified an increased number of ER remnants juxtaposed to mitochondrial membranes, compared to those from the WT samples (Fig. 5). This indicated that GM1 accumulation in the ER membranes fostered the formation of more contact sites between ER and mitochondria. Moreover, MAMs isolated from *β-Gal*^*–/–*^ brains^36,52^ contained substantially higher levels of GM1 than WT MAMs^52^, and the amount was even greater in the purified raft/GEM fractions of the MAMs (Fig. 5). These findings unequivocally proved that GM1 is a normal constituent of these lipid microdomains, albeit present in minuscule amounts, and accumulates in these microdomains from *β-Gal*^*–/–*^ MAMs. These studies have established the existence of rafts/GEMs microdomains within the MAMs and demonstrated that under disease conditions the increased concentration of GM1 at the GEMs facilitates the formation and constitutive activation of a Ca^2+^ pore composed of the IP3R1, VDAC1 and GRP75 (Figs. 2 and 5; Table 1)^52^. Remarkably, the observation that the phosphorylated and active form of the IP3R1 (P-IP3R1) is enriched in the *β-Gal*^*–/–*^ GEMs, and co-precipitates with GM1 (Fig. 5) suggests that GM1 accumulation alters the dynamics of the lipid microenvironment, and favors the partitioning of IP3R1 in an active conformation into these microdomains. Concomitantly, the high levels of VDAC1 and GRP75 observed in the *β-Gal*^*–/–*^ GEMs (Fig. 5), further potentiates the activity of the Ca^2+^ megachannel composed by these proteins. These pathological changes in the composition of the *β-Gal*^*–/–*^ MAMs promotes the continuous Ca^2+^ flux into the mitochondria, which ultimately leads to mitochondria Ca^2+^ overload (Figs. 2 and 3). The net result of these events affects the bioenergetic activities of the mitochondria, and activates the mitochondrial intrinsic apoptotic pathway (Fig. 3)^52^. Signs of mitochondrial dysfunction include mitochondria depolarization, opening of the PTP and mitochondrial membrane permeabilization, which together contribute to mitochondria-mediated apoptosis downstream of GM1 accumulation in the MAMs/GEMs (Fig. 3). Treatment of *β-Gal*^*−/−*^ cells with MBCD, that efficiently extracts GM1 from the MAMs, rescues the opening of the PTP, the dissipation of the potential, and the apoptotic process (Fig. 6). Taken together these studies underscore the significance of the MAMs/GEMs microdomains in preserving physiological ER homeostasis and mitochondrial bioenergetics activity.

## Conclusions and perspectives

In eukaryotic cells membrane-enclosed organelles, albeit individually entrusted to different functions, are highly dynamic and in constant communication with one another (Fig. 1). Their regulated interplay occurs at membrane tethering sites embedded in an active cytoskeletal network, whose formation creates the most efficient setting for the cell to respond quickly to certain physiological or pathological cues. The contact sites between the ER and mitochondria or MAMs are a good example of these functional hubs that control fundamental processes of the corresponding organelles by limiting or enhancing the bidirectional transfer of molecules and ions. The fact that the MAMs embed subdomains enriched in cholesterol and GM1, similar to the lipid rafts of the PM, emphasizes the direct involvement of these constituents in the regulated trafficking of molecules and ions between the two organelles, which can tilt the cell fate towards survival or apoptosis. Thus, it is not surprising that changes in the MAM’s lipid composition/concentration in response to pathological conditions, as in GM1-gangliosidosis, lead to cell death. Many fundamental questions on the biology of the MAMs remain unanswered. For instance, it is still a matter of debate whether variations in the lipid and/or protein signature of the MAMs exist in different cell types or in response to physiologic/pathologic stimuli. What is clear is that specific lipid combinations within the MAMs dictate the recruitment and activity of distinct sets of proteins. It will be of great interest to understand if in other LSDs, like NPC, the failed egression of cholesterol from lysosomal membranes and its accumulation in intracellular membranes also affect the raft/GEM subdomain of the MAMs. If this would be the case, we could anticipate that impaired activity of the MAMs could represent a major contributing factor to disease pathogenesis, especially in lipid storage diseases.
